# Fine particulate matter exposure and sperm DNA fragmentation in US men: a spatial cross-sectional study

**DOI:** 10.1093/humrep/deaf173

**Published:** 2025-09-02

**Authors:** Yuval Fouks, Denis A Vaughan, Pietro Bortoletto, Jeffrey Che-Wei Chang, Daniel Lantsberg, Vivekananda X Datta, Brian McSweeney, Joel David Schwartz, Denny Sakkas

**Affiliations:** Boston IVF—IVIRMA Global Research Alliance, Waltham, MA, USA; Reproductive Services Unit, The Royal Women’s Hospital, Parkville, VIC, Australia; Department of Obstetrics and Gynecology, Beth Israel Deaconess Medical Center, Boston, MA, USA; Department of Obstetrics, Gynecology & Reproductive Biology, Harvard Medical School, Boston, MA, USA; Department of Obstetrics and Gynecology, Beth Israel Deaconess Medical Center, Boston, MA, USA; Department of Obstetrics, Gynecology & Reproductive Biology, Harvard Medical School, Boston, MA, USA; Memorial Sloan Kettering Cancer Center, New York, NY, USA; Reproductive Services Unit, The Royal Women’s Hospital, Parkville, VIC, Australia; Quest Diagnostics, Marlborough, MA, USA; ReproSource Fertility Diagnostics, Marlborough, MA, USA; Quest Diagnostics, Marlborough, MA, USA; ReproSource Fertility Diagnostics, Marlborough, MA, USA; Harvard T.H. Chan School of Public Health, Cambridge, MA, USA; Boston IVF—IVIRMA Global Research Alliance, Waltham, MA, USA

**Keywords:** air pollution, sperm DNA fragmentation, fine particulate matter, male fertility, epidemiology, socioeconomic disparities

## Abstract

**STUDY QUESTION:**

Does exposure to fine particulate matter (PM_2_._5_) impact sperm DNA fragmentation?

**SUMMARY ANSWER:**

Higher PM_2_._5_ exposure was associated with increased sperm DNA fragmentation, with greater effects observed in men of lower socioeconomic status (SES).

**WHAT IS KNOWN ALREADY:**

Environmental air pollutants such as PM_2_._5_ have been linked to adverse reproductive and perinatal outcomes. However, their impact on sperm chromatin integrity remains underexplored, particularly in the context of geographic and sociodemographic modifiers.

**STUDY DESIGN, SIZE, DURATION:**

This was a cross-sectional study including 21 851 semen samples collected between 2005 and 2022 from men undergoing fertility evaluation across multiple US regions.

**PARTICIPANTS/MATERIALS, SETTING, METHODS:**

Semen samples were obtained from men older than 18 years, with testing performed in a single reference laboratory. Exposure to PM_2_._5_ was estimated using validated satellite-derived models and aligned with the 70–80 day spermatogenic window prior to sample collection. Spatial linear mixed-effects models incorporating natural splines and geographic correlation structures were used to assess nonlinear associations between PM_2_._5_ and sperm DNA fragmentation index (DFI), while adjusting for age, SES, population density, and racial composition. Interaction terms were used to evaluate effect modification.

**MAIN RESULTS AND THE ROLE OF CHANCE:**

Higher PM_2_._5_ exposure was associated with increased DFI (estimate = 0.45; *P* = 0.0025), with a clear nonlinear dose–response pattern peaking at ∼11 µg/m³. A significant interaction was observed between PM_2_._5_ and SES (estimate = 0.45; *P* = 0.0148), indicating that men from lower SES areas experienced stronger pollution-related DNA damage. Age remained a strong independent predictor: men ≥50 years showed markedly elevated DFI (estimate = 14.36; *P* < 0.0001).

**LIMITATIONS, REASONS FOR CAUTION:**

The sample was derived from men seeking fertility evaluation and may not represent the general population. ZIP-code level SES and exposure proxies may not reflect to the full extent an individual-level exposures, and residual confounding is possible.

**WIDER IMPLICATIONS OF THE FINDINGS:**

These results underscore the reproductive health consequences of environmental air pollution and its intersection with social inequality. PM_2_._5_ exposure may disproportionately affect sperm chromatin quality in disadvantaged populations; this finding supports targeted environmental and reproductive health interventions. Sperm DNA fragmentation may serve as a biomarker of environmental and social stress.

**STUDY FUNDING/COMPETING INTEREST(S):**

This study was internally funded. V.X.D. and B.M. are employees of ReproSource, which provided laboratory testing, and Quest Diagnostics. No other conflicts of interest were reported.

**TRIAL REGISTRATION NUMBER:**

N/A.

## Introduction

Male infertility accounts for up to 50% of infertility cases in heterosexual couples (WHO, 2021). This contribution is expected to increase in the future due to increasing paternal age (Bellver *et al.*, 2008; Chan and Robaire, 2022; Aitken, 2023) as well as a possible global decline in sperm counts (Hassan and Killick, 2003; Luo *et al.*, 2023; Lewis *et al.*, 2025).

One factor thought to be related to declining sperm counts has been pollution. Pollution, especially fine particulate matter (PM_2_._5_, i.e. fine particulate matter with a diameter of 2.5 μm or smaller), has emerged as a critical environmental factor influencing health outcomes (Lafuente *et al.*, 2016; Carré *et al.*, 2017). PM_2_._5_ particles, due to their small size, can penetrate the respiratory tract and enter the bloodstream, causing systemic inflammation and oxidative stress.

The World Health Organization (2021) recommends an annual PM_2_._5_ mean < 10 µg/m³ to minimize long-term health risks. In the USA, the Environmental Protection Agency (EPA) Air Quality Index classifies annual PM_2_._5_ < 12 µg/m³ as ‘good’ and 12–35 µg/m³ as ‘moderate’, with levels above 35 µg/m³ being linked to increasingly adverse cardiovascular and respiratory outcomes (EPA, 2020).

Recent studies have indicated that exposure to PM_2_._5_ is not only associated with respiratory and cardiovascular diseases but also has potential implications for reproductive health, including male infertility (Nakhjirgan *et al.*, 2023; Ding *et al.*, 2024; Peden, 2024; Gholami Shahrebabak *et al.*, 2025). Several studies or meta-analyses have linked high sperm DNA fragmentation (SDF) with poorer ART outcomes (relative risk (RR) 0.74 implantation; RR 0.83 pregnancy; Ribas-Maynou *et al.*, 2021); (OR 1.68 lower clinical pregnancy rates; Simon *et al.*, 2017), (RR 2.16 miscarriage; Robinson *et al.*, 2012); (RR 0.85 pregnancy; RR 1.57 miscarriage; Deng *et al.*, 2019), (DNA fragmentation index (DFI) outperforming semen parameters; Santi *et al.*, 2018), although an umbrella review by Maghraby *et al.* (2024) has argued that the evidence remains inconsistent.

Despite advancements in male infertility treatments, concerns persist regarding potential compromise of the paternal genome introduced into the oocyte (Sakkas and Alvarez, 2010; Lewis and Kumar, 2015). The sperm DFI is frequently mentioned in the context of sperm assessment but is yet to be fully integrated into clinical use, and its actual significance continues to unfold (Schlegel *et al.*, 2021).

Research has shown that abnormalities in sperm DNA and chromatin can adversely affect reproductive outcomes in both animals and humans (Hales *et al.*, 1992; Robaire and Hales, 2003; Anway *et al.*, 2005; Miska and Ferguson-Smith, 2016). Studies suggest that high levels of DNA damage in sperm are linked to reduced fertility, increased miscarriage rates, and a negative impact on outcomes in ART (Carrell *et al.*, 2003; Zini and Libman, 2006; Bungum *et al.*, 2007; Bungum *et al.*, 2011; Oleszczuk *et al.*, 2016). The mechanisms behind these DNA anomalies are multifaceted and include chromatin dynamics during spermatogenesis, apoptosis in the seminiferous tubules, and oxidative stress induced by reactive oxygen species (ROS) (Sakkas and Alvarez, 2010; Aitken and Curry, 2011).

A number of studies have shown that high pollution levels, particularly long-term exposure to air pollutants, are linked to lower sperm counts, reduced motility, and, in some cases, increased DNA fragmentation. A meta-analysis confirmed significant declines in sperm counts and concentrations in men from highly polluted areas (Rubes *et al.*, 2005; Jurewicz *et al.*, 2018; Zhang *et al.*, 2020; Kleshchev *et al.*, 2021; Cheng *et al.*, 2022).

However, comprehensive studies on the impact of various pollutants on sperm DNA integrity are lacking, with most research focusing on specific pollutants or highly polluted regions. This highlights the need for large-scale, multicenter studies to better understand the effects of pollution on sperm DNA; this is also essential for developing targeted public health interventions.

Given the importance of environmental factors on male fertility, our study focuses on the impact of PM_2_._5_ levels on semen parameters such as DFI and oxidative stress. By employing spatial regression models, we aim to account for confounding by geographical variations and sociodemographic characteristics, analyzing data across different ZIP codes in the USA. This analysis allows us to explore the complex interactions between pollution and male reproductive health and to provide insights into regional differences by accounting for potential geographic proximity dependencies. Understanding the spatial variation and the impact of PM_2_._5_ on sperm quality can help identify regions at higher risk and inform targeted public health interventions.

## Materials and methods

### Study samples

A semen analysis was performed, and DFI, high DNA stainability (HDS), and oxidative stress activity (OSA) values were measured on all samples and has been described in a prior publication by this group (Vaughan *et al.*, 2020). Samples were collected between January 2005 and December 2022. We included all male participants older than 18 who provided semen samples across the USA. A total of 21 851 samples were available for analysis. All data were obtained in a de-identified form, and all samples were processed at a single reference laboratory (ReproSource, (Advanced Semen Report)). The data provided included the fields outlined in Supplementary Table S1. DFI testing was performed uniformly on all consecutive samples received. All assays were run using the sperm chromatin structure assay (SCSA) as described previously (Vaughan *et al.*, 2020), with identical procedures applied to every specimen to minimize indication bias. All semen analyses and SCSA assays were performed in accordance with the 6th edition of the WHO Laboratory Manual (WHO, 2021) and the standards in semen examination where appropriate (Bjorndahl *et al.*, 2022) (Supplementary Table S2).

### Study population and data sources

Participants were excluded if they had missing or invalid ZIP codes, missing or invalid covariates and exposure estimates, or if they did not meet the inclusion criteria for age and health status. This study leveraged data from multiple sources, including clinical records, environmental exposure databases, and socioeconomic datasets.

We included all men aged >18 years who submitted semen samples for fertility evaluation at ReproSource between January 2005 and December 2022. Samples were collected as part of routine fertility work-ups (e.g. infertility or pre-ART assessments). We excluded any specimens with invalid or missing five-digit ZIP codes, missing key covariates (age, DFI, OSA, or HDS), or implausible values (e.g. age <18 or >50). No specific comorbidity or lifestyle exclusion criteria were applied, since our de-identified dataset lacked systematically recorded clinical comorbidity, smoking, or BMI data. All included participants underwent standard DFI testing regardless of underlying health history, provided they met the age and data-completeness requirements.

To relate zip codes to demographic and socioeconomic information, data were derived from the American Community Survey (ACS) (US Census Bureau, 2023), which provides detailed data on population characteristics. The environmental exposure data, specifically PM_2_._5_ levels, were obtained from high-resolution models calibrated at the EPA monitoring stations.

The study was deemed exempt from ethical approval by the New England Independent Review Board (now WCGIRB). Informed consent was waived because the data used were de-identified and collected for research purposes only. The study adhered to the Strengthening the Reporting of Observational Studies in Epidemiology (STROBE) guidelines for cohort studies.

### Data collection and variables

Sperm DFI was measured via the SCSA, as described previously (Vaughan *et al.*, 2020). Briefly, aliquots of washed sperm were treated with acidic detergent, stained with acridine orange, and analyzed by flow cytometry. The DFI reflects the ratio of red (denatured) to total (red + green) fluorescence; values >25–30% denote significant fragmentation. HDS was calculated from the same assay as the proportion of sperm with increased green fluorescence, indicating immature chromatin; HDS >15% was considered elevated. Oxidative stress activity (OSA) was quantified using a chemiluminescence assay (ReproSource kit), measuring ROS in washed sperm suspensions; results are reported in relative light units (RLU) per 10^6^ sperm, with >50 RLU/10^6^ considered abnormal. All assays were performed in a single reference laboratory with standardized protocols.

Supplementary Table S1 provides a legend for the semen parameters and pollution levels analyzed in the study.

Our primary outcome was the impact of PM_2_._5_ levels on sperm DNA integrity, as measured by the DFI assay and reported as DFI and oxidative stress activity (OSA).

We used patient five-digit ZIP codes and date of collection as a linking variable to cross-reference and integrate sociodemographic attributes from external data sources into our spatial mixed model analysis. Specifically, the ACS provided detailed demographic, economic, and housing data. We extracted variables such as income, educational attainment, and population density. Additionally, the Inter-university Consortium for Political and Social Research (ICPSR) (Swanberg, 2017) hosts comprehensive data on social, political, and economic factors. We used these data to obtain additional sociodemographic attributes for our analysis.

A secondary analysis was performed to examine further relationships between the covariates including, age, income, population density, educational attainment, socioeconomic status (SES), and racial group. The census variables tested were as follows: Population Density: average number of people per square mile (or kilometer); Affluence: composite measure of SES (e.g. median income, property values, education levels); and Proportion of Non-Hispanic Black Population: percentage of the population identifying as non-Hispanic Black. All attributes were based on National Neighborhood Data Archive (NaNDA hosted by ICPSR) and the ACS five-year estimates (2013–2017).

The geographic coordinates (latitude and longitude) were recorded and jittered to protect participant privacy. We categorized affluence (SES) into ‘high’ and ‘low’ groups based on a median split, in order to assess pollution impact within strata.

### Exposure assessment

Estimates of PM_2_._5_ concentrations were derived using an advanced spatiotemporal modeling approach that integrates outputs from three distinct machine learning techniques (neural networks, gradient boosting machines, and random forests) each trained on US EPA and ancillary air monitoring data (Di *et al.*, 2016, 2019).

The predictions generated by these models were synthesized using a nonlinear, geographically weighted regression. Model inputs included a diverse set of predictors such as land use variables, outputs from chemical transport models, meteorological data, and satellite-derived measurements. These machine learning frameworks do not impose predefined relationships between predictors and PM_2_._5_, enabling them to naturally account for nonlinear patterns and complex interactions. Model performance was evaluated via 10-fold cross-validation against empirical measurements from national monitoring networks, achieving an *R*^2^ of 0.89 for annual average PM_2_._5_. Daily pollutant estimates were generated on a 1 × 1 km grid and subsequently averaged within ZIP code boundaries to produce daily ZIP code estimates. These were further aggregated to weekly and annual averages for each ZIP code. Participants’ exposures were assigned based on their residential ZIP codes.

Although the study design was cross-sectional, exposure timing was aligned with the presumed spermatogenic window, ∼70–80 days prior to semen collection, to enhance biological plausibility. Specifically, each participant was assigned the ZIP code-level annual mean PM_2_._5_ concentration, serving as a surrogate for chronic exposure during this critical period. This strategy aimed to improve the relevance of the exposure metric while minimizing reliance on short-term fluctuations.

Due to the anonymized nature of the dataset, individual-level identifiers were unavailable, preventing linkage of multiple semen samples to the same participant. Consequently, each semen sample was treated as an independent data point. Given that most men in this cohort underwent DFI testing only once, this approach is unlikely to have substantially influenced the results.

### Framework and statistical analysis

We utilized a spatial regression framework to investigate the relationships between PM_2_._5_ pollution levels and semen parameters, DFI, and OSA. Given the geographical nature of the data, mixed-effects models with spatial covariance structures were employed to account for spatial dependencies and variability between locations. Missing data comprised <1% of zip code records.

#### Mixed-effects models

Mixed-effects models were chosen for their ability to handle both fixed effects (e.g. PM_2_._5_ levels, age groups) and random effects (e.g. variability between locations). To account for spatial autocorrelation, we tested several spatial covariance structures, including Gaussian, Spherical, and Matern. Although the Matern covariance structure was considered for its flexibility, we primarily used the exponential correlation structure, which is a special case of the Matern structure with a smoothness parameter fixed at 0.5. The exponential structure was chosen for its computational simplicity and its ability to model short-range spatial dependencies effectively. The range parameter in the model indicated the extent of spatial correlation. Fixed effects included PM_2_._5_ levels and age groups, while random effects accounted for variability between geographical locations.

This model included pollution as a continuous predictor with covariates such as age, population density, affluence, and racial composition, while accounting for spatial dependencies. A 2.5-unit increase was used to represent a moderate yet realistic change within our observed range in PM_2_._5_ levels, allowing assessment of how incremental pollution increases might impact SDF.

#### Model fitting

Models were fitted using the nlme package in R. To address potential biases from overrepresented regions (e.g. major cities), we applied location-based weighting. Weights were calculated by determining the sample size for each location, and the weight for each location was computed as the ratio of the median (or mean, in a separate analysis) sample size to the sample size at that location.

#### Other analyses employed

Hotspot analysis was performed (Supplementary Fig. S1) using the Getis-Ord statistic to identify regions with significant clustering of high or low values of the semen parameters. This analysis helped visualize spatial patterns and identify potential areas of concern or interest (De Smith *et al.*, 2018).

#### Model evaluation

Model fit was assessed using Akaike Information Criterion (AIC), Bayesian Information Criterion (BIC), and log likelihood values, with lower AIC and BIC values indicating better model fit, while higher log likelihood values suggested a better fit.

A linear mixed-effects model with natural splines was applied to explore the nonlinear relationship between PM_2_._5_ levels and sperm DFI. The model included a natural spline term for PM_2_._5_ (ns(pm25_mean, df = 4)) to capture potential nonlinear dose–response effects on DFI. Additional covariates included age group, population density, affluence, and the proportion of Black residents. Random effects were specified for location, and spatial correlation was modeled using an exponential structure based on geographic coordinates. Weights were applied to account for variations in sample size across locations. Model validity and reliability were assessed using residual diagnostics and confidence intervals.

#### Sensitivity analysis

Sensitivity analysis was conducted to evaluate the robustness of the relationship between PM_2_._5_ levels and DFI under different analytical conditions. The approaches included: (i) varying weight calculations, where different methods for calculating weights were explored, using both the mean and median of sample sizes, to assess the impact of weight calculation on model outcomes, and (ii) threshold identification and categorization. A linear mixed-effects model with natural splines was employed to explore potential inflection points or threshold effects at varying PM_2_._5_ levels. The natural splines approach captured nonlinear trends while avoiding overfitting. Based on these findings, PM_2_._5_ levels were categorized into four exposure groups, low (0–5 µg/m³), moderate (5–10 µg/m³), high (10–15 µg/m³), and very high (>15 µg/m³), to reflect real-world exposure patterns and enhance interpretability.

Bootstrap Resampling was used to assess the stability and variability of model estimates; we applied resampling with 1000 iterations. For each iteration, the dataset was resampled with replacement, maintaining the original sample size. Percentile-based 95% confidence limits (CL) were calculated for all model parameters to provide robust measures of variability and assess the reliability of the findings.

For high-exposure observations, we calculated the 95th percentile cutoff of pm25_mean (17.3 µg/m³) and removed all samples above this threshold (retaining ∼95% of participants). On the restricted dataset, we refitted two mixed-effects models: Null model (Age_Group, popden13_17, affluence13_17, pnhblack13_17, spatial correlation, and a random intercept for Location) and Spline model (same as the null model plus a natural spline term for pm25_mean (df = 4)). We compared model fit via AIC and a likelihood-ratio test (LRT) to assess whether the nonlinear association persisted after excluding the highest 5% of exposures. Predicted DFI curves from both models were plotted across the reduced PM_2_._5_ range, and model coefficients, AIC, LRT statistics, and *P*-values are presented in Supplemental Table S3.

## Results

### Semen parameters and PM_2_._5_ exposure by age group

**Figure 1.**
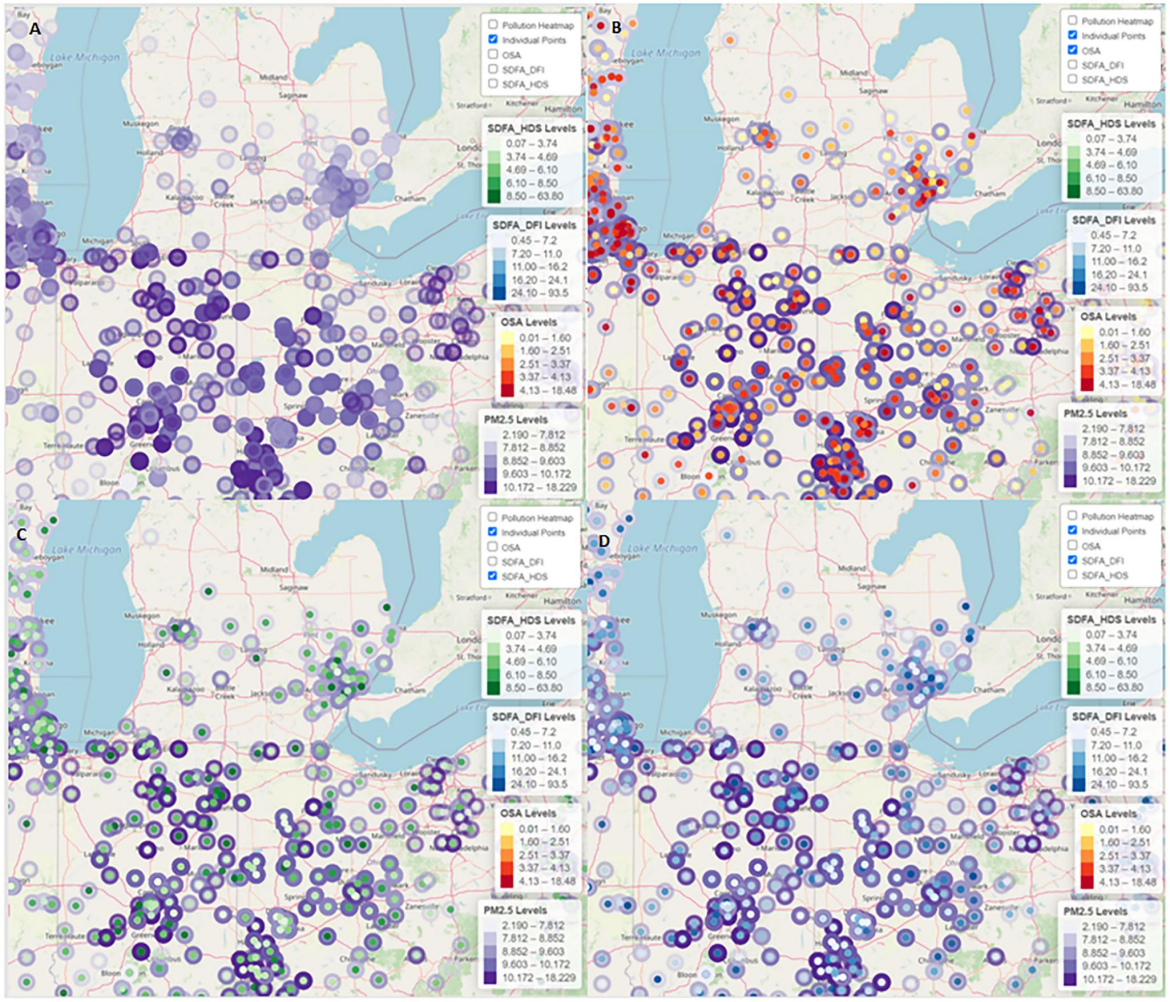
**Pollution and semen parameters across regions.** This figure is part of interactive maps designed for the study, presenting a spatial analysis of fine particulate matter (PM_2_._5_) and semen parameters across regions in the USA. Panel (**A**) maps mean PM_2_._5_ concentrations (light- to dark-purple) with individual sampling sites. Panel (**B**) overlays PM_2_._5_ concentrations with oxidative stress activity (OSA, yellow-to-red). Panel (**C**) overlays PM_2_._5_ concentrations with high DNA stainability (HDS, green-to-blue). Panel (**D**) overlays PM_2_._5_ concentrations with sperm DNA fragmentation index (DFI, light blue to dark blue). All color bars indicate quantitative ranges in the units shown.

Table 1 shows semen parameters and PM_2_._5_ levels by age group. Age effects were measured relative to the reference group (age 18–20). DNA fragmentation increased with age, while HDS decreased. Oxidative stress activity (OSA) levels remained stable with age. PM_2_._5_ exposure varied across different age groups. Figure 1 and Supplementary Fig. S2 present visual distributions of individual DFI data cross-referenced with pollution layers across selected US regions.

**Table 1.** Semen parameters and PM_2_._5_ levels by age group.

Parameter		≤20 (n = 15)	21–30 (n = 3127)	31–40 (n = 13 149)	41–50 (n = 4739)	≥50 (n = 821)	*P*-value
Oxidative stress activity (OSA)	RLU × 10⁶ sperm	3.29 ± 1.86	3.02 ± 1.59	2.98 ± 1.57	2.97 ± 1.59	2.87 ± 1.66	0.147
DNA fragmentation index (DFI)	%	12.37 ± 9.31	14.34 ± 9.64	15.72 ± 10.24	18.20 ± 12.52	23.88 ± 16.93	<0.001
High DNA stainability (HDS)	%	6.95 ± 6.17	6.79 ± 4.81	6.49 ± 4.04	6.18 ± 3.55	6.00 ± 3.77	<0.001
Annual PM₂.₅ mean	µg/m³	8.50 ± 1.36	8.96 ± 1.66	9.01 ± 1.60	9.02 ± 1.72	8.85 ± 1.73	0.021
Days between sample collection and assay	Days	79.20 ± 9.12	77.36 ± 8.23	76.75 ± 7.93	77.11 ± 8.11	77.06 ± 8.09	<0.001

OSA (oxidative stress activity): measures oxidative stress levels in semen, relevant to sperm quality and fertility. DFI (sperm DNA fragmentation index): indicates the percentage of sperm with DNA fragmentation, which can impact fertility. HDS (high DNA stainability): reflects the proportion of sperm with immature chromatin, assessed in sperm chromatin structure assays. PM₂.₅ mean: average concentration of PM_2_._5_ (particulate matter <2.5 μm), a common indicator of air pollution. DFI percent: proportion of sperm showing DNA fragmentation, related to DFI. HDS percent: proportion of sperm with high DNA stainability, related to HDS. DFI cat: categorical grouping of DNA fragmentation index results, potentially classifying them as low, medium, or high. OSA cat: categorical grouping of oxidative stress levels in semen samples.

### SDF and PM_2_._5_ exposure

All reported PM_2_._5_–DFI associations were adjusted for age group. Age remained a strong independent predictor. The spatial covariance structure model revealed that PM_2_._5_ levels significantly increased DFI (estimate = 0.73, *P* < 0.0001). Compared to the reference group (age 18–20), Age Group 21–30 showed a significant increase in DFI (estimate = 6.59, *P* = 0.001), and Age Group 50+ exhibited the largest increase (estimate = 14.41, *P* < 0.0001). Significant increases were also observed for Age Groups 31–40 (estimate = 7.82, *P* = 0.001) and 41–50 (estimate = 9.19, *P* = 0.0002).

### OSA and PM_2_._5_ exposure

The mixed-effects model assessing the impact of PM_2_._5_ and age on OSA (Table 2) showed that PM_2_._5_ had a significant negative effect on OSA (estimate = −0.03, *P* = 0.0382). Using Age Group 18–20 as the reference, Age Group 21–30 showed a significant increase in OSA (estimate = 0.83, *P* = 0.015), as did Age Groups 31–40 (estimate = 0.93, *P* = 0.006), 41–50 (estimate = 0.86, *P* = 0.011), and 50+ (estimate = 0.77, *P* = 0.023).

**Table 2.** Spatial mixed-effects regression analysis of the impact of PM_2_._5_ on sperm DNA fragmentation and oxidative stress across different age groups and geographical locations.

Fixed effect	Estimate (DFI)	Std. error (DFI)	*P*-value (DFI)	Estimate (OSA)	Std. error (OSA)	*P*-value (OSA)
(Intercept)	1.41	2.70	0.60	2.30	0.37	<0.0001
pm25_mean	0.73	0.09	<0.0001	−0.03	0.01	0.038
Age Group 21–30	6.59	2.51	<0.0001	0.83	0.34	0.015
Age Group 31–40	7.82	2.50	0.002	0.93	0.34	0.006
Age Group 41–50	9.19	2.50	<0.0001	0.86	0.34	0.012
Age Group ≥50	14.41	2.51	<0.0001	0.77	0.34	0.024
Population density (popden13_17)	0.000013	0.00013	0.35	−0.000006	0.0000019	0.002
Affluence (affluence13_17)	0.17	1.15	0.87	0.19	0.17	0.25
Proportion non-Hispanic Black (pnhblack13_17)	−7.34	1.90	<0.0001	0.12	0.26	0.65

Age Groups 21–30, 31–40, 41–50, 50+: estimates for different age groups, with Age Group 18–20 as the reference (reflects the increase in DFI as age increases). popden13 17 (population density): influence of population density (per 1000 people per square mile) on DFI and OSA. affluence13 17 (affluence): impact of affluence on DFI and OSA, reflecting socioeconomic factors. pnhblack13 17 (non-Hispanic Black population): influence of the percentage of the non-Hispanic Black population on DFI and OSA.

### Dose–response relationship: PM_2_._5_ and DFI (continuous analysis)

Supplementary Table S3 presents the results of a linear mixed-effects model using natural splines to assess the relationship between continuous PM_2_._5_ exposure and DFI. The spline analysis revealed a nonlinear dose–response trend in which DFI increases with PM_2_._5_ levels, peaking at ∼11 µg/m³. Beyond this value, the curve plateaus, likely due to fewer observations in the higher exposure ranges.

When stratified by PM_2_._5_ quartile (Supplementary Table S4), the proportion of men with DFI >25% increased from 15.0% in Q1 to 17.6% in Q2 and 17.1% in Q3, then fell to 16.6% in Q4. HDS >15% followed the same trajectory (5.9%, 7.0%, 6.9%, and 5.5% across Q1–Q4, respectively). These results confirm that both DFI and HDS peak in the intermediate PM_2_._5_ exposure range before plateauing or declining at the highest levels.

### Dose–response relationship: PM_2_._5_ categories and DFI

Table 3 shows the association between categorized PM_2_._5_ exposure levels and DFI. Compared to the reference group (low PM_2_._5_: ≤5 µg/m³), moderate exposure (5–10 µg/m³) was associated with a significant increase in DFI (estimate = 3.62, *P* = 0.0035), as was high exposure (10–15 µg/m³) (estimate = 3.70, *P* = 0.0034). However, the very high exposure group (>15 µg/m³) showed no significant association (estimate = −2.25, *P* = 0.6314).

**Table 3.** Mixed-effects regression results for the impact of PM_2_._5_ categories and age groups on DFI.

Fixed effect	Estimate	Std. error	*P*-value
(Intercept)	4.44	2.83	0.1176
**PM₂.₅ Category: moderate**	**3.62**	**1.24**	**0.0035**
**PM₂.₅ Category: high**	**3.70**	**1.26**	**0.0034**
PM₂.₅ Category: very high	−2.24	4.68	0.6314
**Age Group: 21–30**	**6.53**	**2.51**	**0.0094**
**Age Group: 31–40**	**7.80**	**2.50**	**0.0019**
**Age Group: 41–50**	**9.19**	**2.50**	**0.0002**
**Age Group: 50+**	**14.36**	**2.51**	**<0.0001**
Population density (popden13_17)	0.00002	0.000014	0.1596
Affluence (affluence13_17)	0.51	1.19	0.6692
**Proportion black (pnhblack13_17)**	**−5.76**	**1.91**	**0.0026**

Association of PM_2_._5_ Exposure Categories, Age Groups, and Covariates with Predicted Sperm DNA Fragmentation Index (SDFA_DFI). This table presents the fixed effect estimates, standard errors, and *P*-values for the linear mixed-effects model analysing the association between PM_2_._5_ exposure, age groups, and other covariates (population density, affluence, and proportion of Black residents). PM_2_._5_ exposure was categorized as follows: low (0 ≤ PM_2_._5_ ≤ 5): represents areas with very low PM_2_._5_ exposure; moderate (5 < PM_2_._5_ ≤ 10): represents areas with moderate PM_2_._5_ exposure; and high (10 < PM_2_._5_ ≤ 15): represents areas with high PM_2_._5_ exposure. Very high (PM_2_._5_ > 15): represents areas with very high PM_2_._5_ exposure. The model accounts for random effects by location and spatial correlation. Significant associations (*P* < 0.05) are in bold.

After removing the ∼5% of samples with PM_2_._5_ >17.3 µg/m³, we refitted both models on the remaining data. The spline model still fit in a comparable way to the covariate-only model (AIC 195 850 vs 196 551; ΔAIC ≈ 702), and a LRT confirmed a highly significant nonlinear PM_2_._5_–DFI relationship (*χ*^2^_4_ = 709.6, *P* < 0.0001).

### Sociodemographic predictors of DFI

Sociodemographic attributes were assessed in relation to DFI outcomes (Tables 3 and 4). Population density was not a significant predictor (*P* = 0.1596). Affluence also did not show a significant effect on DFI (*P* = 0.6692).

**Table 4.** Regression results for the impact of PM_2_._5_, SES, and demographic factors on DFI.

Variable	Estimate	Std. error	*P*-value
(Intercept)	4.16	2.86	0.1461
PM₂.₅ mean	0.45	0.14	0.0025
High SES (affluence)	−4.37	1.77	0.0138
PM₂.₅ × high SES interaction	0.45	0.18	0.0148
Age Group: 21–30	6.59	2.51	0.0088
Age Group: 31–40	7.82	2.5	0.0018
Age Group: 41–50	9.19	2.5	0.0002
Age Group: 50+	14.43	2.51	<0.0001
Population density	0.000014	0.000014	0.3111
Non-Hispanic Black population	−7.1	1.89	0.0002

This table summarizes the regression analysis exploring the relationships between PM_2_._5_ levels, socioeconomic status (SES), and sperm DNA fragmentation index (DFI). The analysis accounts for covariates such as age group, population density, and the percentage of the non-Hispanic Black population.

### Interaction between PM_2_._5_ and DFI and SES

Table 4 summarizes a model incorporating an interaction term between PM_2_._5_ levels and SES. PM_2_._5_ exposure remained significantly associated with increased DFI (estimate = 0.45, *P* = 0.0025). High SES was associated with decreased DFI (estimate = −4.37, *P* = 0.0138). The interaction term between PM_2_._5_ and high SES was significant (estimate = 0.45, *P* = 0.0148), suggesting SES modified the impact of PM_2_._5_ on DFI.

Supplementary Table S5 summarizes the HDS results. HDS was higher in men with moderate and high PM_2_._5_ exposure (5–15 µg/m³) compared to low exposure (≤5 µg/m³; both *P* = 0.036), with no further increase at very high levels (>15 µg/m³; *P* = 0.61).

## Discussion

### Principal findings

This US multi-state study work allows an insight into the impact of air pollution on sperm DNA quality, revealing a significant association between PM_2_._5_ exposure and DNA fragmentation. Our findings demonstrate a significant association between PM_2_._5_ and sperm DNA integrity, underscoring the potential risks of environmental pollution on reproductive health. The inferences align with earlier studies performed in much higher pollution areas (particularly in industrial areas) that have highlighted the effects of air pollution on sperm DFI (Bosco *et al.*, 2018; Vozdova *et al.*, 2022; Calamai *et al.*, 2023).

Our study’s contribution lies in demonstrating that these potentially linked changes to sperm DNA quality are evident even in areas with relatively lower pollution levels, as seen in the USA. This suggests that sperm DNA integrity is highly associated with environmental factors, reinforcing the importance of considering air quality as a factor in reproductive health. These findings highlight the need for assessing how even modest exposure levels might contribute to broader health outcomes. Furthermore, the study encourages future research to explore protective measures that could mitigate the adverse effects of environmental pollutants on male reproductive health.

### Results in the context of what is known

The relationship between air pollution and male reproductive health has largely focused on standard semen parameters such as count, motility, and morphology (Lafuente *et al.*, 2016; Zhang *et al.*, 2020; Kleshchev *et al.*, 2021; Zhao *et al.*, 2022). Meta-analyses have shown mixed results, with some suggesting limited evidence on the effect of pollution on motility and morphology, while others have highlighted a potential association with SDF (Deng *et al.*, 2016; Lafuente *et al.*, 2016; Qian *et al.*, 2022) in relatively polluted areas. Our findings provide a nuanced view, where PM_2_._5_ levels significantly impacted DFI, supporting the notion that chronic exposure to air pollutants may indeed compromise sperm DNA integrity, as observed in high-exposure regions. These results underscore the importance of accounting for regional differences and demographic factors when assessing the impact of pollution on sperm quality.

### Clinical implications

Our analysis also revealed that socioeconomic factors, particularly in combination with environmental exposures, significantly impact sperm health. DFI results indicated disparities linked to SES. Additionally, the interaction between high SES and PM_2_._5_ was significant, indicating that individuals in higher SES groups might experience distinct PM_2_._5_-related effects on sperm DNA integrity. These findings emphasize the importance of sociodemographic factors in public health strategies, as environmental exposures and SES appear to jointly influence reproductive health outcomes.

For OSA, population density was a significant negative predictor (*P* = 0.0021), possibly reflecting stressors associated with urban living. Affluence and non-Hispanic Black population percentages did not significantly affect OSA, suggesting the relationship between SES and oxidative stress may be influenced by additional, unmeasured factors specific to urban settings.

Our findings suggest that sperm DNA quality may be influenced by a complex interplay of air pollution, age, regional exposure, and sociodemographic factors. While PM_2_._5_ significantly predicted higher DFI, these effects varied geographically, emphasizing the need for spatially sensitive analyses. Additionally, future studies should explore the temporal dynamics of exposure, particularly from acute events like wildfire smoke, to better understand how short-term spikes in pollution may impact sperm health. Integrating temporal data with semen analyses could clarify the cumulative versus immediate effects of pollution on reproductive outcomes. The strong influence of age, as demonstrated in our study, further highlights the need for age-stratified analyses, alongside the control of other confounders such as SES and population density. Localized research focusing on the nuances of environmental and social determinants, including wildfire smoke patterns and sociodemographic disparities, will be critical in advancing our understanding of the interplay between environmental exposures and male reproductive health.

### Strengths and limitations

As with all large cross-sectional population studies, this examination of factors influencing sperm DNA quality has a number of strengths and limitations. Although this large study incorporated over 21 000 samples and one laboratory for analysis, the study was limited because samples had some regional biases, and the males being examined were most likely being assessed due to a couple’s interest in seeking fertility treatment. The study utilized clinical records and environmental databases, which may have biases and inaccuracies, although these databases have been part of numerous studies examining other clinical questions (Liu *et al.*, 2023; Wei *et al.*, 2024). PM_2_._5_ exposure estimates were based on ZIP code-level data, and although weighting was introduced in the analysis to avoid bias, the patient locations may not have captured individual exposure accurately. Spatial regression models were also used to account for geographical variability, but unmeasured confounders may still influence the results. Jittering geographic coordinates for privacy could introduce spatial imprecision. There is also always a possible temporal nature to sperm DNA quality, as originally shown by Evenson *et al.* (1999). To counter this, the study adopted a cross-sectional design enhanced with a time-matching component (matching the exposure date with the semen ejaculate date), allowing for a more precise assessment; however, this could be improved in future analysis by exploring longitudinal designs to understand temporal dynamics better. While this study primarily relies on a cross-sectional design, we attempted to temporally align exposure by linking each semen sample to the 70–80 days preceding collection, which corresponds to the full spermatogenic cycle. Notably, our exposure assessment utilized annual average PM_2_._5_ concentrations at the ZIP code level, which serve as a surrogate for relatively prolonged chronic environmental exposure. This approach enhances the relevance of our findings for long-term biological effects. Nonetheless, we acknowledge that more granular, individual-level exposure trajectories over multiple years could better capture cumulative pollution burden. Future longitudinal studies incorporating such time-resolved data may further clarify the chronic impact of air pollution on male reproductive function.

Another limitation was that conventional semen parameters were unavailable for most participants; we could not examine their potential confounding or mediating roles. Future work should include full semen profiles alongside DFI and oxidative stress metrics.

Our analysis did not include individual-level factors such as varicocele status, abstinence interval, smoking history, or body mass index, as these data were not captured in the de-identified clinical database. We acknowledge that the absence of these covariates may lead to residual confounding. Future studies should incorporate these key clinical variables to more fully disentangle PM_2_._5_ effects from other personal risk factors.

Our analysis highlights two other key limitations. The distribution of PM_2_._5_ levels reveals a significantly lower frequency of observations in the highest pollution levels (>12 µg/m³) compared to mid-range levels. This sparse data exemplified in Supplementary Fig. S3, may contribute to statistical instability and flattening of trends at very high pollution levels. Future analyses should include sensitivity tests to assess whether the observed trends persist when extreme PM_2_._5_ levels are excluded or down-weighted. Finally, a potential survivor bias should be considered. Men residing in high-pollution areas with severely impaired sperm quality may be underrepresented in the dataset, either due to infertility, reduced likelihood of seeking fertility evaluation, or other health-related barriers. As a result, the individuals captured in these regions may represent a healthier subset, potentially skewing the data and artificially lowering the average DFI at the highest PM_2_._5_ exposure levels. This selection effect may in part explain the observed plateau or decline in DFI at very high pollution levels and should not be interpreted as a protective effect. When we excluded the top 5% of PM_2_._5_ exposures, the spline-based mixed-effects model still exhibited a peak in predicted DFI at ∼11 µg/m³. Thus, the apparent plateau or decline at very high PM_2_._5_ levels is unlikely to be driven solely by healthy-survivor bias or a few outliers. However, alternative explanations—such as residual confounding that varies across exposure levels (e.g. unmeasured lifestyle or occupational factors), selective participation or differential response among individuals in highly polluted areas, and potential smoothing artifacts inherent to spline modeling—cannot be fully ruled out.

Additionally, because our cohort derives from men seeking fertility evaluation at a private reference laboratory (often self-paying), there may be an inherent socioeconomic bias: even individuals residing in lower-SES ZIP codes could represent a higher-income subset able to afford fertility testing. This selection bias may limit generalizability and could partially confound the observed SES × PM_2_._5_ interaction. Moreover, as participants were fertility patients and thus likely more health-conscious or symptomatic, the results may not generalize to the overall male population. However, because DFI testing is almost exclusively self-ordered and paid out-of-pocket, our cohort closely mirrors the real-world population of men who elect to have DFI measured. Therefore, while our findings do not necessarily apply to all men, they should be generalizable to the broader subset of men undergoing DFI screening.

### Public implications

Broad population studies have long been referenced as the basis of informing policy (Chen *et al.*, 2024; Yang and Umair, 2024; Zheng *et al.*, 2024). The association of PM_2_._5_ levels with SDF reinforces the belief that reducing air pollution remains crucial for overall health. An impact of a change in air pollution diminution was best evidenced overall during Covid where many large cities experienced changes in pollution levels (Ghahremanloo *et al.*, 2022; Patel *et al.*, 2024; Song *et al.*, 2024; Wei *et al.*, 2024). The possible implications pollutant reduction can have on sperm quality were best shown by Calamai *et al.* (2023), who showed that during and after Covid lockdown in Tuscany, there was an associated improvement in sperm motility and DNA quality, suggestive of the potential role of air pollution in male infertility. Policymakers should, therefore, continue efforts to lower environmental pollution, as it may have a general impact on aspects of reproductive health in both males and females. Furthermore, conducting repeated measurements-based studies will help establish causal relationships and improve our understanding of the long-term impacts of age and environmental factors on semen quality. These studies would allow future research to delve deeper into the biological mechanisms underlying age-related increases in DNA fragmentation, including the roles of oxidative stress and DNA repair pathways.

Finally, our findings support evidence that semen quality reflects broader male health. Studies by Eisenberg *et al.* show poor semen parameters often correlate with higher risks of cardiovascular disease, cancer, and other health issues, with adverse sperm results sometimes preceding these conditions (Eisenberg *et al.*, 2014; Eisenberg *et al.*, 2015; Kasman *et al.*, 2020; Del Giudice *et al.*, 2021). Thus, the negative impact of PM_2_._5_ on DFI may indicate not just reproductive risks but also broader health vulnerabilities, underscoring semen analysis as a valuable general health indicator, especially in high-pollution areas.

## Conclusions

The study substantiates the significant impact of male age on DNA fragmentation, reinforcing its role as a recognized determinant of sperm DNA integrity, together with established attributes such as age and chemotherapy. While PM_2_._5_ levels showed a significant effect on DFI, their influence was comparatively less pronounced when accounting for age and other biological determinants. This suggests that environmental factors, while having an impact, may have a more subtle role in isolation but could act as an exacerbating ‘second hit’ in the presence of underlying biological vulnerabilities, further compromising sperm quality. Additionally, our findings highlight the multifactorial nature of male infertility by incorporating socio-demographic attributes.

These observations underscore the importance of considering both spatial and sociodemographic variations in reproductive health research, emphasizing the need for comprehensive models that integrate environmental, biological, and socio-demographic factors to better understand their combined impact on semen quality.

## Supplementary Material

deaf173_Supplementary_Figure_S1

deaf173_Supplementary_Figure_S2

deaf173_Supplementary_Figure_S3

deaf173_Supplementary_Table_S1

deaf173_Supplementary_Table_S2

deaf173_Supplementary_Table_S3

deaf173_Supplementary_Table_S4

deaf173_Supplementary_Table_S5

## Data Availability

This study utilized de-identified data sourced from clinical records and publicly available databases. Researchers interested in accessing the data can contact Y.F. at fouksy@thewomens.org.au.
