# Supplementary material for: Fine particulate matter exposure and sperm DNA fragmentation in US men: a spatial cross-sectional study
Source: Hum Reprod. 2025 Sep 2;40(10):1850–9. doi: 10.1093/humrep/deaf173 (PMC12491671; doi:10.1093/humrep/deaf173)
Supplement: deaf173_Supplementary_Figure_S1 [file deaf173_supplementary_figure_s1.pdf]

Human Reproduction, 2025, 1–1
https://doi.org/10.1093/humrep/deaf173
Supplementary Data

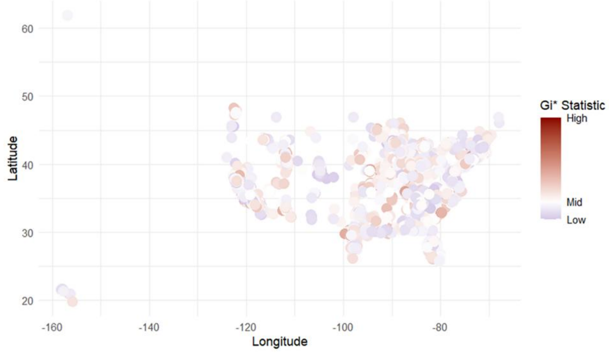

Supplementary Figure S1. Hotspot analysis with Getis-Ord. Hotspot analysis of $PM_{2.5}$ impact on DFI using Getis-Ord Gi* statistic. This map illustrates the spatial distribution of the Getis-Ord Gi* statistic across the USA, representing hotspots and coldspots of $PM_{2.5}$ impact on the DFI. The Gi* statistic is color-coded. Red shades: indicating high Gi* values, suggesting areas where $PM_{2.5}$ levels are significantly associated with higher DFI values (hotspots). Blue shades: indicating low Gi* values, suggesting areas where $PM_{2.5}$ levels are significantly associated with lower DFI values (coldspots). Mid shades: representing areas with moderate or no significant association between $PM_{2.5}$ levels and DFI. The geographic coordinates (latitude and longitude) are plotted on the *x* and *y* axes, respectively, covering various regions across the USA. This visualization helps identify regions with significant positive or negative spatial auto-correlation in the impact of $PM_{2.5}$ on sperm DNA fragmentation.
