# Supplementary material for: Fine particulate matter exposure and sperm DNA fragmentation in US men: a spatial cross-sectional study
Source: Hum Reprod. 2025 Sep 2;40(10):1850–9. doi: 10.1093/humrep/deaf173 (PMC12491671; doi:10.1093/humrep/deaf173)
Supplement: deaf173_Supplementary_Figure_S2 [file deaf173_supplementary_figure_s2.pdf]

OXFORD

human reproduction

Human Reproduction, 2025, 1–1

https://doi.org/10.1093/humrep/deaf173

Supplementary Data

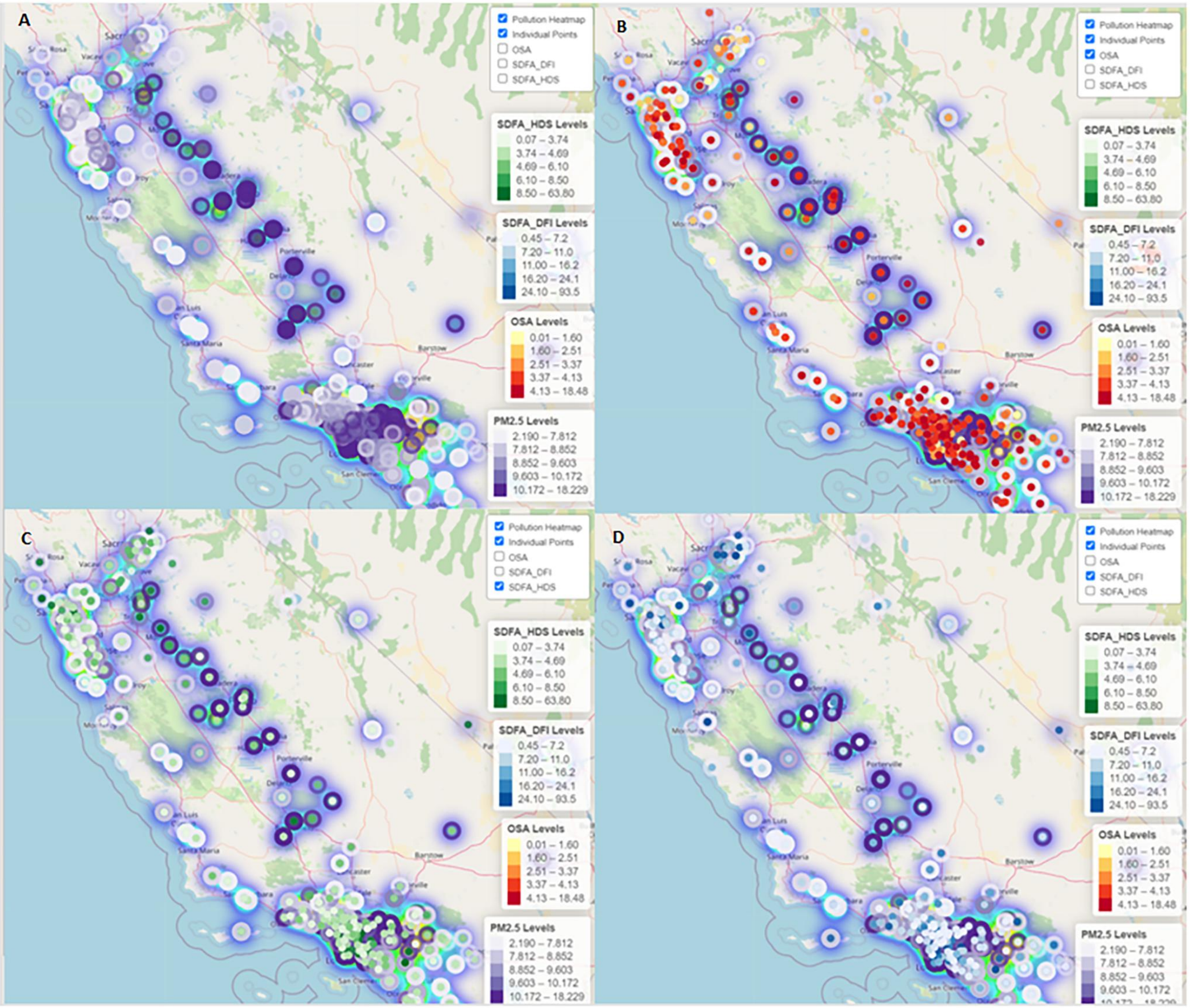

Supplementary Figure S2. Spatial distribution of air pollution and semen-quality markers in the US West Coast region. This figure is part of interactive maps designed for the study, presenting a spatial analysis of fine particulate matter ($PM_{2.5}$) and semen parameters in the Western USA. Panel (**A**) maps mean $PM_{2.5}$ concentrations (light- to dark-purple) with individual sampling sites. Panel (**B**) overlays $PM_{2.5}$ concentrations with oxidative stress activity (OSA, yellow-to-red). Panel (**C**) overlays $PM_{2.5}$ concentrations with high DNA stainability (HDS, green-to-blue). Panel (**D**) overlays $PM_{2.5}$ concentrations with sperm DNA fragmentation index (DFI, light blue to dark blue). All color bars indicate quantitative ranges in the units shown.
