# Supplementary material for: Fine particulate matter exposure and sperm DNA fragmentation in US men: a spatial cross-sectional study
Source: Hum Reprod. 2025 Sep 2;40(10):1850–9. doi: 10.1093/humrep/deaf173 (PMC12491671; doi:10.1093/humrep/deaf173)
Supplement: deaf173_Supplementary_Figure_S3 [file deaf173_supplementary_figure_s3.pdf]

Human Reproduction, 2025, 1–1
https://doi.org/10.1093/humrep/deaf173
Supplementary Data

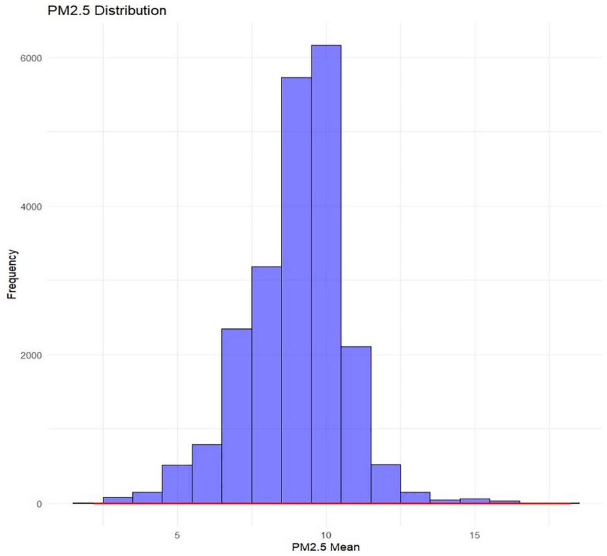

Supplementary Figure S3. Distribution of mean ambient PM assigned to semen samples included in the analysis. Bars show frequency counts in 0.5-µg m categories.
