# Supplementary material for: Fine particulate matter exposure and sperm DNA fragmentation in US men: a spatial cross-sectional study
Source: Hum Reprod. 2025 Sep 2;40(10):1850–9. doi: 10.1093/humrep/deaf173 (PMC12491671; doi:10.1093/humrep/deaf173)
Supplement: deaf173_Supplementary_Table_S1 [file deaf173_supplementary_table_s1.pdf]

OXFORD
human reproduction

Human Reproduction, 2025, 1–1
https://doi.org/10.1093/humrep/deaf173
Supplementary Data

Supplementary Table S1. Legend notes for semen parameters and pollution.

Abbreviation	Full term	Description
OSA	Oxidative stress activity	A measure often used in fertility studies to determine the level of oxidative stress in semen, which can affect sperm quality.
DFI	Sperm DNA fragmentation index	Measures the percentage of sperm with DNA fragmentation, an indicator of damage that can impact fertility.
HDS	High DNA stainability	A measure used in sperm chromatin structure assay (SCSA) tests to quantify the proportion of sperm with immature chromatin or excess DNA stainability.
pm25 mean	Average concentration of $PM_{2.5}$	Refers to the average concentration of $PM_{2.5}$, fine particulate matter with a diameter of <2.5 μm, used as a common measure of air pollution.
DFI percent	DNA fragmentation percentage	Measures the percentage of sperm that exhibit DNA fragmentation, similar to DFI.
