# Supplementary material for: Fine particulate matter exposure and sperm DNA fragmentation in US men: a spatial cross-sectional study
Source: Hum Reprod. 2025 Sep 2;40(10):1850–9. doi: 10.1093/humrep/deaf173 (PMC12491671; doi:10.1093/humrep/deaf173)
Supplement: deaf173_Supplementary_Table_S2 [file deaf173_supplementary_table_s2.pdf]

Human Reproduction, 2025, 1–1
https://doi.org/10.1093/humrep/deaf173
Supplementary Data

Supplementary Table S2. Compliance with WHO and semen-analysis standards.*

	Checklist item	Compliance	Location
Patient description	Inclusion/exclusion criteria clearly defined	Yes	Methods, p. 4
Collection method	Abstinence period specified	Yes	Methods, p. 4
Specimen handling	Time from collection to analysis stated	Yes	Methods, p. 5
Concentration	Sperm concentration measured	N/A	—
Motility	Motility categories defined	N/A	—
Morphology	Morphology criteria and staining method described	N/A	—
DFI assay	DFI cutoff defined	Yes	Methods, p. 5,
HDS assay	HDS cutoff defined	Yes	Methods, p. 5
OSA assay	N/A	Yes	Methods, p. 5

* See WHO Semen Standards, 2021 and Bjorndahl *et al.*, 2022.
