# Supplementary material for: Fine particulate matter exposure and sperm DNA fragmentation in US men: a spatial cross-sectional study
Source: Hum Reprod. 2025 Sep 2;40(10):1850–9. doi: 10.1093/humrep/deaf173 (PMC12491671; doi:10.1093/humrep/deaf173)
Supplement: deaf173_Supplementary_Table_S3 [file deaf173_supplementary_table_s3.pdf]

OXFORD

human reproduction

Human Reproduction, 2025, 1–1
https://doi.org/10.1093/humrep/deaf173
Supplementary Data

Supplementary Table S3. Dose–response analysis of $PM_{2.5}$ on SDFA_DFI.

Variable	Effect (estimate)	Standard error	P-value
$PM_{2.5}$ spline terms			
Spline 1 (ns(pm25_mean, df = 4)1)	61.99	2.45	<0.001
Spline 2 (ns(pm25_mean, df = 4)2)	36.68	1.83	<0.001
Spline 3 (ns(pm25_mean, df = 4)3)	129.08	6	<0.001
Spline 4 (ns(pm25_mean, df = 4)4)	−9.79	5.19	0.059
Age groups			
Age 21–30	7.62	2.48	0.002
Age 31–40	8.76	2.47	<0.001
Age 41–50	10.17	2.47	<0.001
Age 50+	15.31	2.48	<0.001
Covariates			
Population density (popden13_17)	0.00001	0.00001	0.31
Affluence (affluence13_17)	−1.95	1.3	0.135
Proportion Black (pnhblack13_17)	−7.27	1.97	<0.001

Dose–response analysis of $PM_{2.5}$ on SDFA_DFI using a mixed-effects model with splines.
This table summarizes the fixed-effects results of the mixed-effects model exploring the nonlinear dose–response relationship between $PM_{2.5}$ exposure (modeled with splines) and sperm DNA fragmentation index (SDFA_DFI). Key covariates such as age, population density, affluence, and proportion Black are also included. Significant associations are highlighted, showing how $PM_{2.5}$ and other factors impact SDFA_DFI. Random effects account for spatial correlation and clustering by location.
