# Supplementary material for: Fine particulate matter exposure and sperm DNA fragmentation in US men: a spatial cross-sectional study
Source: Hum Reprod. 2025 Sep 2;40(10):1850–9. doi: 10.1093/humrep/deaf173 (PMC12491671; doi:10.1093/humrep/deaf173)
Supplement: deaf173_Supplementary_Table_S4 [file deaf173_supplementary_table_s4.pdf]

OXFORD
human reproduction
Human Reproduction, 2025, 1–1
https://doi.org/10.1093/humrep/deaf173
Supplementary Data

Supplementary Table S4. Proportion of men with abnormal DFI and HDS by $PM_{2.5}$ quartile.

$PM_{2.5}$ quartile	% DFI abnormal	% HDS abnormal
Q1 (lowest)	15.0	5.9
Q2	17.6	7.0
Q3	17.1	6.9
Q4 (highest)	16.6	5.5

$PM_{2.5}$ quartiles are based on the distribution of annual mean pm25_mean within the study cohort (each quartile ≈ 25% of participants).
DFI abnormality defined as DFI >25%; HDS abnormality defined as HDS >15%, based on standard SCSA thresholds.
Percentages represent the proportion of men in each quartile exceeding the respective thresholds.
