# Supplementary material for: Fine particulate matter exposure and sperm DNA fragmentation in US men: a spatial cross-sectional study
Source: Hum Reprod. 2025 Sep 2;40(10):1850–9. doi: 10.1093/humrep/deaf173 (PMC12491671; doi:10.1093/humrep/deaf173)
Supplement: deaf173_Supplementary_Table_S5 [file deaf173_supplementary_table_s5.pdf]

Human Reproduction, 2025, 1–1
https://doi.org/10.1093/humrep/deaf173
Supplementary Data

Supplementary Table S5. Fixed-effects estimates for HDS (SDFA_HDS) from categorical $PM_{2.5}$ exposure model.

Predictor	Estimate	SE	P-value
(Intercept)	4.26	1.09	0.0001
$PM_{2.5}$: moderate (5–10)	0.96	0.46	0.0358
$PM_{2.5}$: high (10–15)	0.98	0.47	0.0355
$PM_{2.5}$: very high (>15)	−0.92	1.80	0.6110
Age: 21–30	1.96	0.98	0.0444
Age: 31–40	1.23	0.97	0.2045
Age: 41–50	0.75	0.97	0.4413
Age: 50+	1.35	0.98	0.1664
Population density	0.000011	0.0000052	0.0287
Affluence index	0.056	0.434	0.8987

Reference categories: $PM_{2.5}$ low (≤5 μg/m^3); Age group ≤20 years. Model details: linear mixed-effects model with a random intercept for ZIP code (Location), exponential spatial correlation (based on geographic coordinates), and inverse-variance sampling weights. $PM_{2.5}$ exposure classification: based on annual mean $PM_{2.5}$ at the time of semen collection, categorized as low (≤5), moderate (5–10), high (10–15), and very high (>15 μg/m^3). HDS: high DNA stainability (SDFA_HDS) measured as percent of sperm showing immature chromatin. Interpretation: estimates reflect the adjusted difference in mean HDS relative to the reference category.

© The Author(s) 2025. Published by Oxford University Press on behalf of European Society of Human Reproduction and Embryology.
This is an Open Access article distributed under the terms of the Creative Commons Attribution License (https://creativecommons.org/licenses/by/4.0/), which permits unrestricted reuse, distribution, and reproduction in any medium, provided the original work is properly cited.
Human Reproduction, 2025, 00, 1–10
https://doi.org/10.1093/humrep/deaf173
Original Article
